# Supplementary material for: Closed-loop stimulation of temporal cortex rescues functional networks and improves memory
Source: Nat Commun. 2018 Feb 6;9:365. doi: 10.1038/s41467-017-02753-0 (PMC5802791; doi:10.1038/s41467-017-02753-0)
Supplement: Supplementary file 1 — Supplementary Information [file 41467_2017_2753_MOESM1_ESM.pdf]

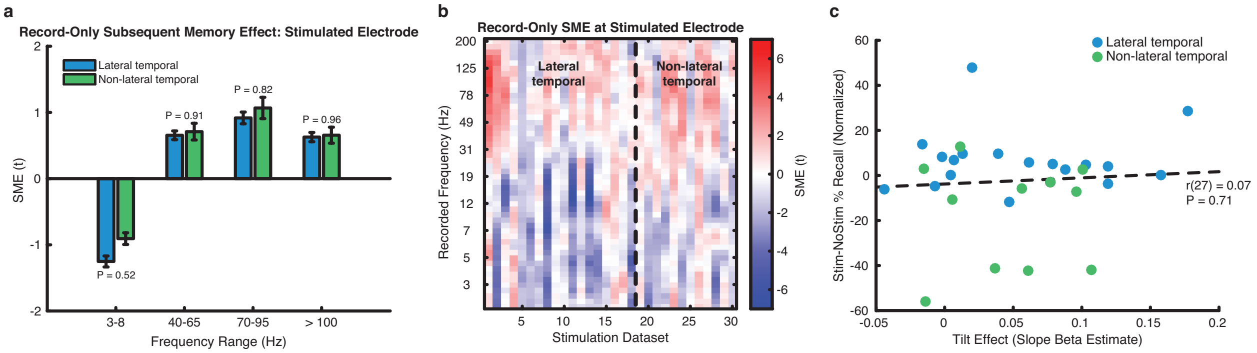

Supplementary Figure 1 | Subsequent memory effect (SME) at stimulated electrode pair. (**a,b**) The subsequent memory effect during the record-only sessions at the stimulated electrode pair did not differ between the Lateral temporal and Non-lateral temporal groups (two-sample t-tests). Stimulated electrodes demonstrated a spectral tilt in the SME: positive SMEs at high frequencies (> 40 Hz) and negative SMEs at low frequencies (3—8 Hz). (**c**) We computed the slope of the frequency spectrum to measure the spectral tilt at the stimulated electrode pair and found this measure did not correlate with the effect of stimulation on memory performance in later closed-loop sessions (Pearson correlation). Data are presented as mean ± s.e.m.

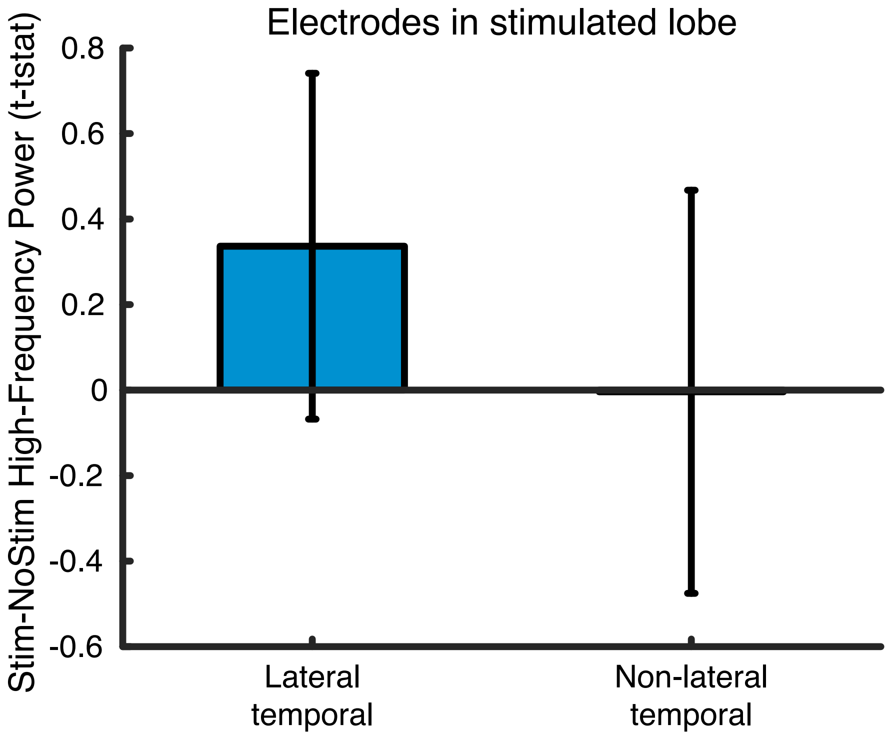

Supplementary Figure 2 | Evoked high-frequency power following stimulation. Within electrodes that were placed in the same lobe and hemisphere as the stimulation target, we extracted wavelet power for 1000~ms windows following stimulation and matched NoStim epochs. We averaged power over time and computed unpaired t-tests within each electrode and wavelet frequency in the high-frequency range (70--200~Hz). We then averaged these t-statistics across electrodes and across subjects to derive an aggregated estimate of the effect of stimulation on high-frequency power near the stimulation target site. Across subjects, high-frequency power was not reliably modulated by stimulation in the same lobe as the stimulation target ($P_{LatTemp}$ = 0.40; $P_{NonLatTemp}$ = 0.99; $P_{LatTemp-NonLatTemp}$ = 0.61, N_{LatTemp} = 18, $N_{\mathrm{NonLatTemp}}$ = 11). Data are presented as mean ± s.e.m.

Supplementary Table 1 | Patient Information

Subject	Site	Gender	Age	Ictal Onset	Task	Stimulation Parameters: Target	Hem	Dur (ms)	Freq (Hz)	Amp (mA)	Stim Prec	NoStim Prec
1	J	F	40	L	FR	PRC	L	500	25	0.50	0.26	0.27
2	J	M	45	R	FR	aINS	R	500	200	0.50	0.27	0.3
3	D	F	36	NA	FR	MTG	L	500	200	1.00	0.35	0.34
4	E	F	53	L	FR	CA1	L	500	50	1.00	0.45	0.48
5	T	M	45	L	FR	MTG	L	500	10	0.50	0.11	0.08
6	D	M	38	L	FR	DLPFC	L	500	200	1.00	0.19	0.29
7	J	M	20	L	FR	SMG	L	500	200	0.25	0.15	0.15
8	E	M	44	L/R	FR	STG	L	500	10	1.00	0.4	0.38
					FR	MTG	L	500	200	1.00	0.47	0.5
9	T	M	25	L	FR	MTG	L	500	200	1.00	0.29	0.27
10	P	M	36	R	FR	ITG	R	500	10	1.75	0.26	0.25
11	M	F	29	R	FR	SFG	R	500	50	0.50	0.19	0.17
					FR	IPL	R	500	200	0.75	0.11	0.17
12	T	F	25	L	FR	MTG	L	500	200	0.75	0.42	0.48
13	T	M	37	L	FR	MTG	L	500	200	0.75	0.23	0.22
14	E	F	42	L	FR	STS	L	500	200	0.75	0.06	0.06
15	J	F	56	L	FR	ITG	L	500	200	0.50	0.27	0.17
16	E	M	48	L	FR	ITG	L	500	200	1.00	0.05	0.05
17	J	F	51	L	FR	STS	L	500	100	0.50	0.39	0.4
					CatFR	STS	L	500	100	0.50	0.55	0.53
18	D	F	41	NA	CatFR	TP	L	500	50	2.00	0.27	0.42
19	T	M	63	L/R	CatFR	MTG	L	500	100	1.00	0.33	0.31
20	P	F	61	L	CatFR	DG	L	500	200	1.00	0.14	0.24
21	D	F	57	NA	CatFR	STG	L	500	200	1.25	0.14	0.13
22	P	F	52	L	CatFR	MTG	L	500	200	0.50	0.32	0.33
					CatFR	MTG	L	500	200	0.50	0.23	0.2
23	T	F	44	R	CatFR	MTG	L	500	200	0.75	0.31	0.3
24	J	M	33	R	CatFR	MTG	R	500	200	0.75	0.38	0.4
25	J	F	57	L	CatFR	MTG	L	500	200	0.75	0.29	0.26

Site: D: Dartmouth-Hitchcock Medical Center, E: Emory University Hospital, J: Thomas Jefferson University Hospital, M: Mayo Clinic, P: Hospital of the University of Pennsylvania, T: University of Texas Southwestern Medical Center

Task: FR: free recall, CatFR: categorized free recall

Ictal Onset: L: Left, R: Right, NA: Not Reported/Undetermined

Target: STG: superior temporal gyrus, STS: superior temporal sulcus, MTG: middle temporal gyrus, ITG: inferior temporal gyrus TP: temporal pole, PRC: perirhinal cortex, CA1: hippocampal subfield CA1, DG: dentate gyrus, SMG: supramarginal gyrus, IPL: inferior parietal lobule, aINS: anterior insula, SFG: superior frontal gyrus, DLPFC: dorsolateral prefrontal cortex

Stim Prec: Mean proportion of words recalled on stimulated lists
NoStim Prec: Mean proportion of words recalled on unstimulated lists
